# Scrivener’s Spasm Cured by Division of Muscles

**Published:** 1842-09

**Authors:** 


*Scriveners' Spasm cured by Division of Muscles.* By Professor Stromeyer.—The disease which may be called scriveners' spasm, and consists of permanent contraction of the long flexor of the thumb, is not very rare. Several cases have been published in Germany by Albers, Heyfelder, and Kopp; and in France by Cazenave and David. The chief characteristic of this affection is an absolute incapability of using the pen, for writing, although the strength and motions of the hand remain unimpaired for all other purposes. Some authors regard it as a species of spasm; others, as depending on paralysis; it is generally permanent, but occasionally appears at intervals, and then is commonly brought on by long continued use of the pen. This affection, though apparently slight, is most obstinate, and resists every method of treatment that has hitherto been employed against it. The following cases, which we abridge considerably, had baffled the skill of Professor Stromeyer, until he had recourse to division of the flexor muscle, which was attended with complete success.

Case I.—J. M., a public writer at Hanover, labored under this disease during two years. A celebrated physician had tried every kind of remedy without avail. Whenever he began to write, the muscles of the ball of the thumb were seized with spasm, which compelled him to desist, but the spasms did not appear at any other time. M. Stromeyer also tried a variety of means ineffectually, and then divided the small flexor muscles of the thumb. This likewise failed, and the sensibility of the palmar surface of the thumb was destroyed. It was now clear that the action of writing depended upon the long flexor, but the patient refused to permit any more operations to be performed on him.

Case II.—The subject of this case had suffered under the disease for fifteen years, when first seen by M. Stromeyer. The rigidity of the muscles of the ball of the thumb was not, however, present, but the last phalanx of the thumb became suddenly flexed, whenever the patient attempted to write or play on the piano.

The long flexor was not permanently contracted, nor did it impede any other motions of the hand. From the deep situation of the muscle it was not easy to divide it separately. M. Stromeyer bent the first phalanx strongly to a right angle, and at the same time turned the thumb as much out as possible; he then passed a very small, curved tenotome underneath the tendon, and divided it. The sensibility of the thumb was very considerably diminished after the operation, but was restored, on the dorsal aspect the next day, and on the palmar aspect within a fortnight. The natural power of moving the thumb, also, returned at the latter period, and the patient was able to write or play on the piano without the slighest return of the spasm.—*N. Y. Lancet,* from *Arch. Gen. de Med.*

